# Developments in forensic DNA analysis

**DOI:** 10.1042/ETLS20200304

**Published:** 2021-04-01

**Authors:** Penelope R. Haddrill

**Affiliations:** Centre for Forensic Science, Department of Pure and Applied Chemistry, University of Strathclyde, Glasgow, U.K.

**Keywords:** DNA profiling, forensic biology, forensic genetics

## Abstract

The analysis of DNA from biological evidence recovered in the course of criminal investigations can provide very powerful evidence when a recovered profile matches one found on a DNA database or generated from a suspect. However, when no profile match is found, when the amount of DNA in a sample is too low, or the DNA too degraded to be analysed, traditional STR profiling may be of limited value. The rapidly expanding field of forensic genetics has introduced various novel methodologies that enable the analysis of challenging forensic samples, and that can generate intelligence about the donor of a biological sample. This article reviews some of the most important recent advances in the field, including the application of massively parallel sequencing to the analysis of STRs and other marker types, advancements in DNA mixture interpretation, particularly the use of probabilistic genotyping methods, the profiling of different RNA types for the identification of body fluids, the interrogation of SNP markers for predicting forensically relevant phenotypes, epigenetics and the analysis of DNA methylation to determine tissue type and estimate age, and the emerging field of forensic genetic genealogy. A key challenge will be for researchers to consider carefully how these innovations can be implemented into forensic practice to ensure their potential benefits are maximised.

## Introduction

Since its first use in a criminal case in 1987, the analysis of DNA from biological evidence has revolutionised forensic investigations. The intervening three decades have seen significant advancements in terms of the discrimination power, speed, and sensitivity of DNA profiling methods, as well as the ability to type increasingly challenging samples [1–3]. The establishment of databases of offender and crime scene profiles, and of population allele frequencies, have permitted the identification of suspects from crime scene samples and the development of statistical frameworks for evaluating DNA evidence [1,2]. Recent years have seen the expansion of the number of loci included in short tandem repeat (STR) typing kits and standardisation of core loci across jurisdictions, allowing for greater cross-border sharing of DNA profiling data [4,5]. When a recovered profile matches one found on a DNA database, or generated from a suspect, DNA evidence can thus be extremely powerful.

**Figure 1.**
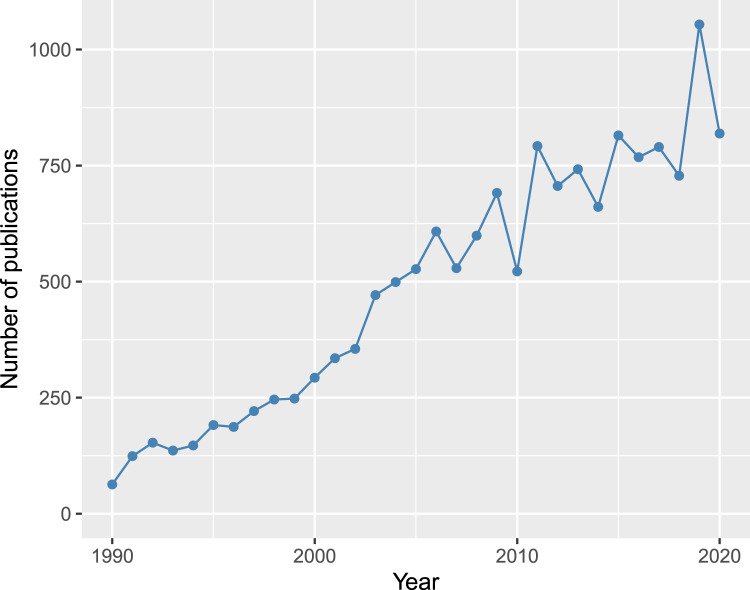
Number of publications returned in a search of www.scopus.com for ‘forensic AND DNA’ in the title, abstract or keywords, for the years 1990 to 2020.

However, when no profile match is found, when the amount of DNA in a sample is too low, or the DNA too degraded to be analysed, traditional STR profiling may be of limited value. The introduction of novel techniques and technologies into the criminal justice system is slow, but new methodologies are being developed that enable the analysis of these challenging samples, and that can generate intelligence about the donor of a biological sample [3,5]. The last few years have seen a rapid expansion in the field of forensic genetics (and now forensic genomics), demonstrated by growing numbers of publications in the field over the last two decades (Figure 1). An exhaustive review of this whole field is beyond the scope of a single article, and so this review seeks to provide an overview of some of the most important recent advances in the forensic analysis of DNA.

## Massively parallel sequencing

Massively parallel sequencing (MPS) technologies, frequently referred to as next-generation sequencing (NGS) technologies, have revolutionised the biological sciences by their ability to generate millions of sequencing reads in a single run. Despite only relatively recently being adopted in the forensic field, the use of MPS for forensic applications has expanded rapidly in the last few years [6,7]. Whilst MPS has permitted high-throughput sequencing of the whole genomes of a huge variety of organisms, forensic applications have used a more targeted approach, including an initial PCR-amplification of a set of target markers prior to MPS of the resulting amplicons [8]. There are two main technology platforms used for forensic applications of MPS, the Illumina sequencing-by-synthesis method, and Thermo Fisher's semiconductor-based Ion Torrent sequencing [9]. A variety of kits are available for use on each platform, targeting different forensically relevant markers, and a growing number of studies have validated and/or evaluated these kits for forensic use (reviewed in [9–11]). For example, the Precision ID Globalfiler™ NGS STR Panel and Precision ID Identity/Ancestry Panels use the Ion S5 system to sequence STR and single nucleotide polymorphism (SNP) markers [12,13], and Promega's PowerSeq® system sequences autosomal and Y-STRs on the Illumina MiSeq [14]. The Verogen ForenSeq™ DNA Signature Prep Kit runs on the MiSeq FGx™ Forensic Genomics System, which uses Illumina technology to sequence a combination of autosomal STRs, Y-/X-STRs, and identity SNPs, and can be expanded to include phenotype- and ancestry-informative SNPs [15,16]. Kits have also been developed to target part or all of the mitochondrial genome [17–19].

**Figure 2.**
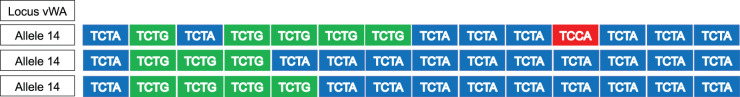
Nucleotide sequence of three alleles detected at the vWA locus. All three of these alleles would be classified as 14 alleles on the basis of their length but determining their nucleotide sequence allows them to be discriminated.

This ability to target large numbers of different marker types into a single assay is one of the key advantages of MPS methods, increasing discrimination power and of particular benefit when analysing the often-limited DNA in forensic samples [20]. Another major advantage of MPS technology is that it detects nucleotide sequence variation in the targeted markers, including variants in STR repeat regions and flanking sequences [21]. This permits discrimination of alleles that would be indistinguishable using capillary electrophoresis length-based typing (Figure 2), a feature that also has advantages for interpretation of complex mixed profiles [22]. MPS also improves results for low level and degraded DNA samples, as a result of the shorter amplicons compared with standard STR profiling [23,24].

There are a number of barriers to widespread adoption of MPS for forensic applications, including variable performance of some markers in terms of coverage and locus imbalance [11], and susceptibility to PCR inhibitors when compared with standard STR typing kits [25,26], but these are rapidly being overcome. Development of a standardised nomenclature system, which captures sequence variation in MPS-generated STR alleles whilst maintaining compatibility with existing CE-based STR data in national DNA databases, is a particular challenge, but recommendations are now in place to address this [27–30]. The availability of frequency data for alleles detected using MPS is also increasing with the publication of datasets from populations worldwide [31–35]. The costs of MPS are decreasing all the time and with the development of bioinformatics tools to analyse the large volume of complex data produced, implementation of MPS technologies into forensic workflows is becoming realistic. Although MPS methods have not yet been widely implemented in casework, with wider applications in other areas of forensic analysis (e.g. RNA sequencing, epigenetics, forensic DNA phenotyping; see below) these technologies are likely to become indispensable tools for the forensic community.

## DNA mixture interpretation

**Figure 3.**
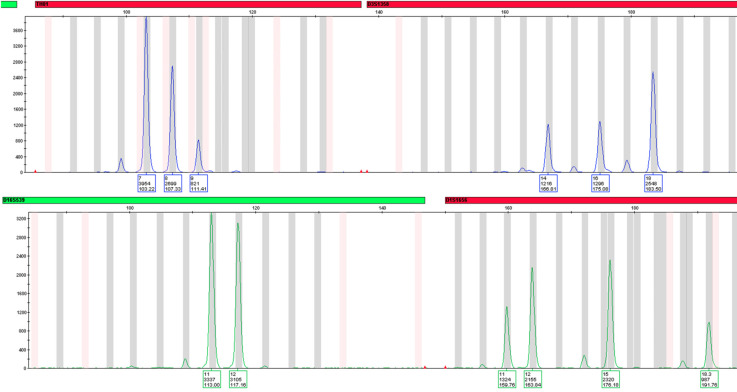
Electropherogram showing part of a mixed DNA profile at four STR loci. Across a whole profile, interpretation of the varying number of peaks and peak heights can become very complex, even with a small number of contributors.

Interpretation of DNA profiles containing contributions from multiple donors is much more complicated than single source profiles (Figure 3), not only because of the potential number of alleles present in the profile, but also because such profiles are often low-level with complicating features such as allele drop-out/drop-in and heterozygous imbalance [36]. The increasing sensitivity of STR profiling techniques means that the recovery of mixed DNA profiles has become more common, not only from samples where mixtures might be expected (e.g. sexual offence samples), but also from low quality/quantity samples recovered from handled items [37]. Such samples often produce complex mixtures, with large numbers of contributors and no individual who can be assumed to be present in the mixture [38].

This increasing complexity of mixed profiles has called for increasingly complex methods of mixture interpretation, and there has been a move away from relatively simple methods that ranged from determining whether an individual could be excluded as a potential contributor to a mixture, to the use of likelihood ratio methods that estimated the most likely genotype combinations of contributors to a mixture, the more complex of which used some of the information contained within profile peak heights [39,40]. This has led to the development of mixture interpretation methods using probabilistic frameworks, incorporating probabilities of allele drop-out and drop-in, modelled from validation and empirical data [41]. These probabilistic genotyping methods are broadly categorised as semi-continuous, which do not utilise peak height information or model artefacts such as stutter, and continuous, which do [42]. The complexity of the statistical calculations involved in these methods is such that specialised software is required to carry out these analyses, and there are a variety of programmes now available for this purpose (summarised in [5]). The ability of these programmes to analyse mixtures previously considered too complicated for interpretation has seen rapid uptake by forensic laboratories, and publication of studies reporting the developmental and internal validation of different probabilistic genotyping software packages, as well as guidelines for their use by a number of regulating bodies [43–48].

The software packages that implement probabilistic genotyping methods are highly complex, and developers have urged forensic laboratories to ensure their analysts have a good understanding of the concepts underlying the methods and that they remain involved in the interpretation of profiles and critical evaluation of the mixture analysis [36,49]. Concerns have been raised over variation in the output of probabilistic genotyping methods, some due to subjective decisions made by the user, some due to variability inherent in the methods [50,51]. Some countries have seen extensive debates over the admissibility of probabilistic genotyping methods in court and whether methods have gained general acceptance in the community, but the widespread implementation of these methods into forensic laboratories around the world suggests they have [42]. These methods also provide significant promise for the interpretation of MPS data that can uncover greater complexity in mixed profiles by identifying sequence differences between alleles that would be indistinguishable by length (Figure 2).

## Body fluid identification

The ability to identify the presence of a specific body-fluid can be extremely valuable to an investigation, providing crucial information on the activities involved in an incident, particularly if it means that a DNA profile can be linked to a specific biological source. All of the presumptive/confirmatory tests currently used to identify some (but not all e.g. vaginal material, menstrual blood) body fluids have limitations, including a lack of sensitivity and specificity, and a requirement to carry out multiple tests that destroy limited samples [52]. This has led to interest in the analysis of RNA in body fluid stains, particularly given RNA can be co-extracted with DNA, allowing parallel production of a DNA profile alongside body fluid testing [53].

Identification of body fluids using RNA profiling is based on the principle that although DNA content is the same in most cell types, RNA differs depending on cell type and function. The production of RNA is therefore tissue-specific, such that each body fluid has a specific gene expression pattern. The presence of tissue-specific RNA types in a sample can therefore indicate the presence of specific body fluids [54]. Research in this area has focused on large-scale screens for differentially expressed RNAs followed by the development of PCR-based assays to target individual or small numbers of markers. Many of these assays employ reverse transcription endpoint (RT-PCR) or quantitative real-time (RT-qPCR) PCR [55], which have the benefit of compatibility with existing technologies in forensic laboratories, although increasingly studies are utilising the power of MPS for identification and analysis of tissue-specific RNAs [56–59].

Initial assays focused on identifying body fluid-specific messenger RNA (mRNA) markers, and development of multiplexes indicating the presence of single or multiple body fluid types, the latter of which is particularly useful when analysing mixed samples (reviewed in [55,60]). However, the susceptibility of mRNAs to degradation has limited their application to forensic samples, and mRNA assays also suffer from limitations including variation in sensitivity and specificity and interpretational challenges [61–64]. More recently, focus has been on micro RNAs (miRNAs) as alternative markers for body fluid identification [65]. Many of these regulatory RNAs, which target mRNAs for degradation or silencing, also show tissue-specific expression, and have the benefit of increased stability compared with mRNA as a result of their smaller size and incorporation into a protein complex within the cell [66]. A variety of miRNAs have been identified as potential markers for forensically relevant body fluids, and although it is unlikely any miRNAs are specific to single body fluid types, a number of assays have been developed that incorporate panels of multiple differentially expressed miRNAs that appear to identify specific body fluids [67–73]. Although miRNA-based assays suffer from some of the same challenges as mRNA-based assays, particularly in terms of interpretation, miRNAs have great potential as body fluid markers [67,68,70]. Further study to identify the best sets of miRNAs to unambiguously identify different body fluids and detailed validation of the resulting assays may give the forensic community a reliable test for the identification of body fluids [65]. Micro RNA markers also hold promise for other forensic applications, including estimating the time of deposition of body fluid stains [74,75] and the post-mortem interval [76].

## Forensic DNA phenotyping

When standard STR profiling fails to advance an investigation because no match to a known suspect or DNA database is found, any information that can assist in identifying the donor of the sample would be very valuable. This has led to the development of tests that predict externally visible characteristics (EVCs) from DNA samples, which can provide intelligence leads to investigations, narrowing the pool of potential suspects [77]. The ability to predict an individual's appearance also has utility in missing persons cases and in disaster victim identification. More widely, forensic DNA phenotyping (FDP) is considered to encompass the prediction of EVCs, inference of bio-geographic ancestry, and the estimation of age using epigenetic markers [78,79].

FDP techniques have developed from many decades of research identifying SNPs that are statistically associated with particular characteristics, via genome-wide association studies [80]. From this, small sets of SNPs have been identified that can be typed in PCR multiplexes and analysed using statistical models that predict EVCs of interest with high accuracy. By far the most advanced and successful of these relate to the prediction of human pigmentation traits [77]. The genetics of physical traits is often complex, with the expression of many traits controlled by variation at a large number of genes, as well as environmental factors [81]. However, human pigmentation traits are influenced by a relatively small number of genes compared with other traits, and it is these pigmentation traits that have been the focus of FDP, principally eye and hair colour and, more recently, skin colour [77].

A number of test systems have been developed for the prediction of human pigmentation traits, including the forensically validated IrisPlex [82,83], HIrisPlex [84,85], and HirisPlex-S [86,87] assays, which predict broad categories of eye, hair, and skin colour by analysing 6, 24, and 41 SNPs, respectively. Inclusion of additional SNPs and improvements in prediction models means that these pigmentation traits can now be predicted with good accuracy, usually expressed using a measure known as the AUC (area under the receiver operating characteristic curve), which can take values from 0.5 (random prediction of the characteristic) to 1.0 (accurate prediction) [77]. For example, the most recent IrisPlex model for eye colour prediction gives accuracies of 0.95 for brown, 0.94 for blue, and 0.74 for intermediate (e.g. non-blue and non-brown) eye colours, reflecting the fact that intermediate eye colours are predicted with lower accuracy as the genetic variants responsible for these colours are less well understood. This resulted in an average eye colour prediction accuracy of 84%, or 93% when only blue and brown categories were included [85]. Similarly, the HIrisPlex model results in hair colour prediction accuracies of 0.92 for red, 0.85 for black, 0.81 for blond, and 0.75 for brown hair colour, giving an average hair colour prediction accuracy of 73% [85]. The accuracy of hair prediction is influenced by the phenomenon of hair darkening with age, which can lead to the prediction of a lighter hair colour than the observed phenotype, and this predominantly affects individuals who are categorised as having brown hair but predicted to be blond [84]. More recently, the accuracy of the HIrisPlex-S skin colour prediction model has been assessed using both 3 and 5 skin colour categories, resulting in AUC values of 0.97 for light, 0.96 for dark-black, and 0.83 for light skin colour categories, and 0.97 for dark-black, 0.87 for dark, 0.74 for very pale, 0.73 for intermediate, and 0.72 for pale skin colour categories [86,87].

The systems described above use multiplex PCR followed by multiplex single-base extension using SNaPshot chemistry, which is limited in terms of the number of SNPs that can be typed in a single multiplex. More recently, the ability of MPS technologies to analyse very large numbers of genetic markers in a single run, even at low levels of input DNA, has been exploited to develop both commercial and custom assays that predict ancestry and/or EVCs [88,89]. For example, researchers in the VISAGE Consortium have combined the 41 SNPs in the HIrisPlex-S system with 115 SNPs that provide information about bio-geographical ancestry to generate an assay that can be run on different MPS platforms [90,91]. VISAGE, the VISible Attributes through GEnomics Consortium, is an EU-funded collaborative research program established in 2017 to work towards the provision of intelligence information about an individual's appearance, age, and ancestry from DNA recovered in the course of investigations (http://www.visage-h2020.eu). The consortium also has a focus on the complex legal, regulatory, and ethical issues surrounding the prediction of EVCs from DNA for forensic purposes, which in many countries is not currently subject to any specific legislation [92,93]. VISAGE researchers and others also continue to make progress on the development of systems to predict other EVCs, including eyebrow colour [94], stature [95], skin features such as freckles and tanning [96], and further hair-related phenotypes such as head hair shape [97] and age-related hair darkening [98].

## Epigenetics and DNA methylation analysis

In addition to the information encoded within the sequence of DNA bases in the genome, the DNA molecule carries an additional layer of information in the form of chemical modifications of nucleotides and chromatin-related proteins [99]. Broadly defined as epigenetic changes, these modifications alter patterns of gene expression via a variety of mechanisms and have been shown to have a role in the regulation of key cellular processes, with epigenetic errors being associated with diseases such as cancer [100]. The addition of a methyl group (-CH_3_) to the 5′ position of cytosine residues in the human genome, primarily those found in cytosine-guanine dinucleotides (known as CpG sites), is one of the most widely studied epigenetic modifications, and observations of differential methylation patterns with age and across tissue types has led to interest in forensic applications of DNA methylation analysis [101]. Research in this area has focused on estimation of the age of the donor of a DNA sample and the identification of tissue-type for body fluids and other forensically relevant biological samples [102–105], although DNA methylation analysis has a range of other forensic applications including the discrimination of monozygotic twins [105] and the determination of smoking status [106].

DNA methylation plays a crucial role in cell differentiation, such that CpG sites are differentially methylated in different tissues [100]. This tissue-specificity has been successfully exploited for the development of methylation-based assays using a range of technologies [103,104]. Numerous studies have described epigenetic markers for the identification of forensically relevant tissue-types, including blood, semen, saliva, vaginal material and menstrual blood, (e.g. [107–114]), some of which pose more significant challenges than others [115–117]. A key benefit of identifying tissue type from the analysis of DNA rather than RNA is that this may provide a link between an STR profile and the corresponding tissue type, given both types of information come from the same molecule [79,103].

The ability to predict the chronological age of an unknown individual from a DNA sample could provide extremely useful intelligence to investigators, particularly in combination with the prediction of EVCs, many of which can vary with age [103]. A number of authors have identified CpG sites where methylation level is correlated with age and built age-prediction models targeting small numbers of sites that can be incorporated into assays to estimate age with high accuracy, using technologies such as pyrosequencing, single base extension using SNaPshot chemistry, and EpiTyper (e.g. [118–120]). Universal age-related markers would have significant benefits in terms of developing models to predict age across multiple tissues, but the highest age-prediction accuracy has been seen in tissue-specific models [102,104,121–123]. These have mainly focused on whole blood [124–128], with some studies on other tissues such as saliva and semen [115,129–132]. A number of MPS-based targeted methylation assays for age prediction have also now been developed, overcoming the multiplexing limitations of previous technologies and permitting analysis of multiple CpG sites in a single highly sensitive assay [104,127,133,134]. These age prediction assays estimate age with high accuracy, measured in terms of the mean absolute deviation (MAD) between the estimated and chronological age, with many assays providing prediction accuracies of ±3–4 years [79,103].

## Genetic genealogy

Since the high-profile arrest in 2018 of Joseph DeAngelo as a suspect in the Golden State Killer investigation, attention has focused on the applications of genetic genealogy in a forensic context [135,136]. Whilst familial searching of forensic DNA databases has been effectively used to identify close (first/second degree) relatives of suspects via the detection of allele sharing in STR profiles, genealogists can identify much larger numbers of more distant relatives (third to ninth degree) by detecting stretches of DNA in the genome that are identical by descent, indicating common ancestry [137]. This is achieved by exploiting huge genetic datasets amassed by individuals taking direct to consumer (DTC) genetic tests for the purposes of genealogical research. These tests type hundreds of thousands of autosomal SNP variants, the results of which are then shared on large public platforms such as GEDmatch (https://www.gedmatch.com/) that allow testers to identify potential relatives [138,139]. Searching of these online platforms using profiles generated from samples recovered in criminal investigations may identify relatives of the potential perpetrator, and further genealogical research may lead to the identification of a suspect whose DNA can then be recovered and compared with crime samples [140].

The size of these public databases of genetic information are such that one study estimated 60% of searches were likely to find a relative at a distance of third cousin or closer, and 15% second cousin or closer, indicating that a database covering only 2% of a target population would include a third cousin match for 99% of the population ([141], see also [142]). The vast majority of people who have taken DTC genetic tests are US citizens of European ancestry, and their over-representation in genealogical databases means the chances of finding relative matches are significantly higher for this population [140,141]. However, there is increasing interest in other populations, both in the uptake of DTC genetic testing by the public and the mining of this data by law enforcement. Since the arrest of DeAngelo and a number of other notable success stories, law enforcement agencies worldwide have begun to see the potential in this approach for identifying the distant relatives of suspects [138].

The approach has sparked concerns about data privacy and ethics, as a result of surreptitious law enforcement searches of public databases, although the majority of platforms that are accessible to law enforcement agencies now either offer consumers the option to opt-out if they do not want their data being included in these types of searches, or explicitly require them to opt in [140,143]. There are ongoing concerns about sharing and privacy of genetic data and the legality of these types of search, as well as the ethics of individuals who have not taken a genetic test being exposed to attention from investigators because a relative has [137,144–146]. There are also no validation studies of genealogical techniques for forensic use [138,139], but the techniques are used only to generate intelligence leads in investigations, often when they have been cold for many years, and any leads would always be verified using standard STR profiling [140]. With the recent acquisition of GEDmatch by forensic genomics company Verogen (https://verogen.com/gedmatch-partners-with-genomics-firm/), along with the launch of a kit specifically designed for genealogical applications (https://verogen.com/products/forenseq-kintelligence-kit/), it seems likely that these methods will become commonplace in investigations.

## Conclusions

This review highlights some of the recent key developments in the rapidly expanding field of forensic genetics, but there are many other exciting areas of research that could not be covered here. For example, methodological developments in DNA extraction [147], direct PCR [148], and rapid/at-scene processing of samples on portable devices (reviewed in [5]), the application of MPS technologies to new marker types such as microhaplotypes [149,150], analysis of non-human DNA in the form of human and environmental microbiomes [151,152], and the use of third-generation sequencing devices in forensic DNA analysis [9,11] represent just some of the current and future developments in the field. It will be crucially important that researchers consider how to harness the innovations produced by this dynamic field to ensure their implementation into forensic practice.

## Summary

- The analysis of DNA from biological material recovered in the course of a criminal investigation can provide very powerful evidence, however when there is no match between the recovered profile and a DNA database or suspect the evidence may be of limited value.
- The rapidly expanding field of forensic genetics research has introduced various novel methods that enable the analysis of challenging forensic samples, and that can generate intelligence about the donor of a biological sample.
- This article reviews some of the most important advances in the field, including the application of massively parallel sequencing, advancements in DNA mixture interpretation, body fluid identification using RNA profiling, forensic DNA phenotyping, epigenetics and DNA methylation analysis, and genetic genealogy.
- A key challenge will be to ensure that the benefits of these novel technologies can be maximised by implementing them into forensic practice.

## Open Access

Open access for this article was enabled by the participation of University of Strathclyde in an all-inclusive *Read & Publish* pilot with Portland Press and the Biochemical Society under a transformative agreement with JISC.

## Abbreviations

DTC: direct to consumer
EVCs: externally visible characteristics
FDP: forensic DNA phenotyping
MPS: massively parallel sequencing
NGS: next-generation sequencing
RT: reverse transcription
SNP: single nucleotide polymorphism
STR: short tandem repeat
